# Transnasal endoscopic orbital decompression

**DOI:** 10.22336/rjo.2017.35

**Published:** 2017

**Authors:** Răzvan Hainăroșie, Irina Ioniță, Cătălina Pietroșanu, Silviu Pițuru, Mura Hainăroșie, Viorel Zainea

**Affiliations:** * ”Carol Davila” University of Medicine and Pharmacy, Bucharest, Romania; ** “Prof. Dr. Dorin Hociotă” Institute of Phonoaudiology and Functional ENT surgery, Bucharest, Romania

**Keywords:** orbit, decompression, endoscopy

## Abstract

Transnasal endoscopic orbital decompression is a viable alternative for external orbital decompression. Today, it can be performed due to the great development of endoscopic technology and specific endoscopic surgical instruments. It requires strong anatomic knowledge and skilled hands.

We present our experience with endoscopic orbital decompression focusing on the surgical technique used in our department. The surgical maneuvers must be firm and gentle protecting the local risk elements and respecting the orbital content.

## Introduction

Orbital decompression was first reported in 1911 by Dollinger, a lateral decompression technique being proposed. Naffziger proposed an orbital roof decompression in 1931. This type of approach was used when managing bilateral disease, and when neurosurgical assistance needed it. Still, the orbit communication with the intracranial content leads to pulsating proptosis. Sewall described the medial decompression of the orbit in 1936. He described the entire removal of the medial wall of the orbit. The orbital content prolabated into the ethmoid cavity. In 1950, Hirsch used the technique described by Lewkowitz and performed an inferior orbitotomy using the transantral Caldwell-Luc approach, removing the inferior orbital wall. The orbital content prolabated into the maxillary sinus. Using a transantral Caldwell-Luc approach, Walsh and Ogura decompressed both the medial and the inferior wall of the orbit in the same surgery.

Using the endoscope, Kennedy imagined the endoscopic approach of the medial wall of the orbit. Michel popularized the technique later in 2001.

The development of the optical technology used in rigid endoscopes and the advancement in optical video chip will provide the surgeon with the right tools to perform the orbital decompression under endoscopic guidance. 

Levine proposed special instruments designed for orbital decompression.

The indications for orbital decompression are the following [**1**,**2**]: 

- ethmoiditis with orbital complications

- ethmoidal mucoceles

- orbital hematoma - trauma or endoscopic sinus surgery

- Graves orbitopathy

- exophthalmos

- exposure keratopathy

- diplopia

- optic neuropathy

- tumors located medial to the optic nerve

- sinonasal or skull base tumors with orbital compartment extension

From an ethical point of view, there are two ways to obtain the informed consent depending on the patients’ general health status. When dealing with a scheduled surgery, the patient needs to be thoroughly presented the surgical plan with its advantages, disadvantages, risks, benefits, possible complications and prognostic, and afterwards the informed consent can be obtained. When facing an emergency situation, the informed consent is not a priority, the surgical and medical gestures have to be performed to save the patient’s life or prevent permanent damages. After the surgery, an emergency report will be written to justify the absence of the informed consent. 

## Materials and method

We analyzed the cases that underwent endoscopic orbital decompression in the IIIrd ENT and Head and Neck Surgery Department of “Prof. Dr. Dorin Hociota” Institute of Phonoaudiology and Functional ENT Surgery in the period 2007-2010. The patients who underwent purely endoscopic orbital decompression were included in the study. In one case, inferior wall orbital decompression was associated to the medial decompression of the orbit.

**Table 1:** Endoscopic orbital decompression

Orbital decompression	Number of patients
Ethmoiditis with orbital complications	19
Ethmoidal mucoceles	14
Orbital hematoma - trauma/ endoscopic sinus surgery	5/12
Optic neuropathy	4
Graves orbitopathy	3
Tumors located medial to the optic nerve	2
Sinonasal tumors with orbital compartment extension	27

The technique used was performed under general anesthesia with orotracheal intubation. Pieces of cotton moisturized in adrenaline solution were mounted along the inferior turbinate and into the medial meatus.

If a major septal deviation was encountered, septoplasty was performed to achieve a good exposure of the ethmoidal bulla.

After 5 minutes, we gently medialized the medial turbinate. The uncinate process and the bulla ethmoidalis were exposed. Inferior uncinectomy was performed. The surgeon had to assess the insertion of the uncinate process preoperatively to avoid harming the orbital contents when uncinectomy was performed. 

**Fig. 1:**
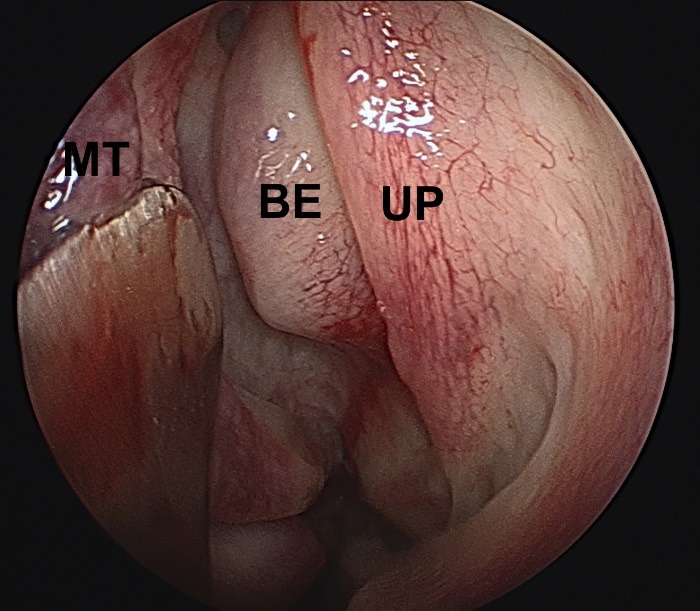
Inspecting the middle meatus. UP – uncinate process; BE – bulla ethmoidalis; MT – middle turbinate

Medial meatotomy was performed; if needed meatotomy orifice could be enlarged, and inferior wall decompression could be conducted.

Anterior ethmoidectomy was done by removing the ethmoidal cells until the lamina papyracea was reached. Lamina papyracea represents the insertion of the medial turbinate on the lateral wall of the nasal cavity. It separates the anterior ethmoid from the posterior one. 

Posterior ethmoidectomy was continued until the skull base was reached. The procedure can be completed when sphenoidotomy is needed, in addition, a total sphenoethmoidectomy was achieved. 

**Fig. 2:**
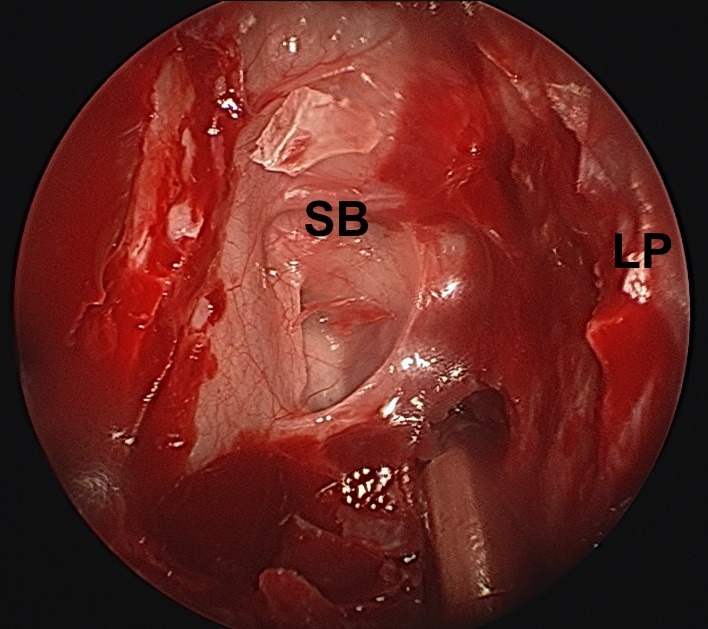
Ethmoidal cavity inspection after anteroposterior ethmoidectomy; LP – lamina papyracea; SB - skull-base

The medial wall of the orbital cavity was exposed widely. The medial wall of the orbit was penetrated with a particular pyramidal tip instrument. The lamina papyracea was removed by using special dissectors with aspiration.

**Fig. 3:**
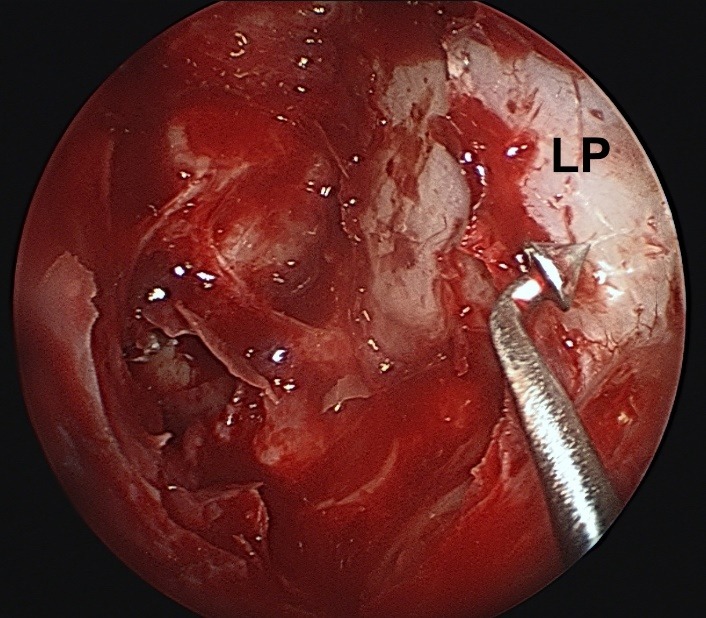
Exposing the medial orbital wall

**Fig. 4:**
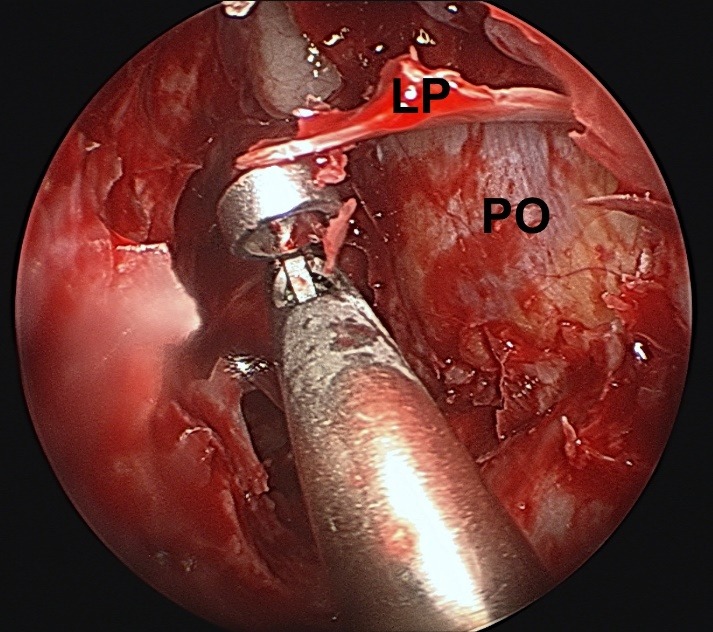
Elevating the bone of the medial orbital wall; LP - lamina papyracea; PO -periorbit

The periorbit was exposed and two or three horizontal incision lines were performed with a blunt tip endoscopic knife. One or two sliding of fascia had to be preserved in patients without optic neuropathy to protect the medial rectus muscle to minimize postoperative diplopia.

**Fig. 5:**
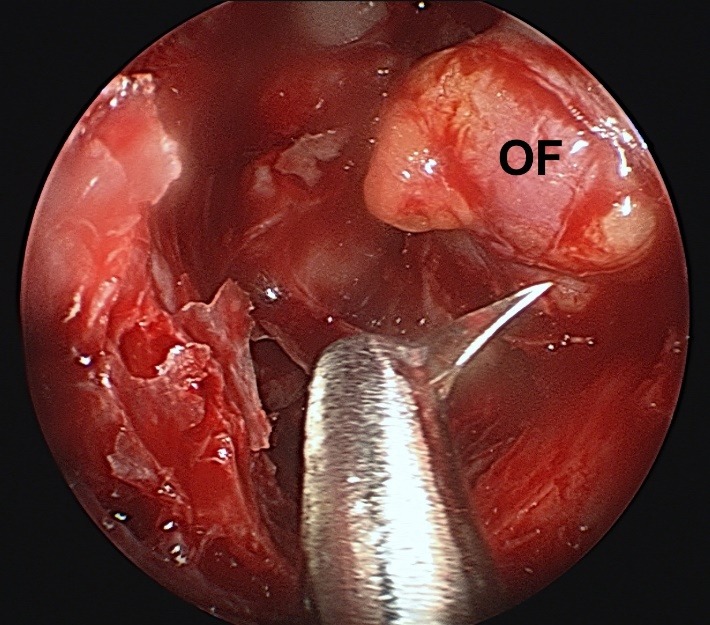
Elevating the bone of the medial orbital wall; LP - lamina papyracea; PO -periorbit

## Result and discussion

Medial orbital decompression was performed successfully. Still, in Graves orbitopathy, we considered that a 360-degree decompression is the gold standard [**6**,**7**].

The orbit is a cone-shaped compartment made up of bony walls circumferentially and the orbital septum anteriorly. The intact orbital septum can manage pressures up to 50 mmHg. The term “orbital compartment syndrome” was presented in 1990 by Kratky. Increased pressure in the intraorbital compartment may compromise the vascularisation of the optic nerve that will lead to irreversible blindness [**3**,**4**]. 

If the blood flow in the posterior ciliary arteries, central retinal artery, or vasa nervorum of the optic nerve is obstructed, optic neuropathy may appear due to ischemia. In orbital abscesses or orbital hematoma, the orbit must be decompressed in the first 8 hours [**5**]. 

## Conclusions

Endoscopic medial orbital decompression is a safe and reliable technique. Advanced anatomic knowledge is necessary to perform the decompression safely. If needed, it can completed with inferior decompression, which can also be performed endoscopically. 

The procedure must be performed by surgeons who can also perform medial canthotomy or decompress the orbit externally. The endoscopic approach offers an excellent visualization and control of the skull base area and orbital apex.

**Acknowledgement**

All the authors have equally contributed to this paper.
